# Dynamic CTA-Based Whole-Brain Arterial-Venous Collateral Assessment for Predicting Futile Recanalization in Acute Ischemic Stroke

**DOI:** 10.14336/AD.2025.0540

**Published:** 2026-06-08

**Authors:** Ruoyao Cao, Yao Lu, Wei Li, Fan Yu, Shen Hu, Kunpeng Chen, Guoxuan Wang, Chengkan Sun, Qingfeng Ma, Miao Zhang, Juan Chen, Jie Lu

**Affiliations:** ^1^Department of Radiology and Nuclear Medicine, Xuanwu Hospital, Capital Medical University, Beijing, China.; ^2^Beijing Key Laboratory of Magnetic Resonance Imaging and Brain Informatics, Beijing, China.; ^3^Department of Radiology, Beijing Hospital, National Center of Gerontology, Beijing, China.; ^4^Department of Neurology, Beijing Hospital, National Center of Gerontology, Beijing, China.; ^5^Department of Neurosurgery, Beijing Hospital, National Center of Gerontology, Beijing, China.; ^6^Department of Neurology, Xuanwu Hospital, Capital Medical University, Beijing, China.

**Keywords:** CTA, Collateral Circulation, Futile Recanalization, Stroke

## Abstract

Futile recanalization is a recognized challenge in acute ischemic stroke (AIS) patients after endovascular treatment (EVT). Our purpose was to develop and validate a predictive model for futile recanalization after EVT by integrating arterial-venous collateral assessment with clinical parameters. This study included 392 AIS patients with acute anterior circulation large vessel occlusion who underwent EVT (March 2016-June 2024). Patients were stratified into training (n = 160), internal validation (n = 69), and completely independent external validation (n = 163) cohorts collected from a separate medical center. Predictors were identified using Boruta algorithm and LASSO regression. Multiple machine learning models were evaluated through discrimination, calibration, and decision curve analyses, with SHAP analysis for feature importance. Three independent predictors were identified: age (OR: 1.06, 95% CI: 1.02-1.11), whole-brain arterial collateral status (OR: 0.30, 95% CI: 0.18-0.50), and whole-brain venous collateral status (OR: 0.78, 95% CI: 0.67-0.90). The model demonstrated excellent discrimination in the training cohort (AUC: 0.914, 95% CI: 0.866-0.963), internal validation cohort (AUC: 0.918, 95% CI: 0.844-0.991), and notably maintained robust performance in the completely independent external validation cohort (AUC: 0.755, 95% CI: 0.678-0.832). Calibration plots showed good agreement between predicted and observed outcomes. SHAP analysis further confirmed the importance of arterial and venous collateral status assessments. The integration of whole-brain arterial-venous collateral assessment with clinical parameters shows potential value in predicting futile recanalization after EVT. This model, validated across multiple cohorts, may provide additional information to support clinical decision-making.

## INTRODUCTION

Futile recanalization, a concept first proposed by Hussein et al. in 2010, is defined as successful vessel recanalization [modified Thrombolysis in Cerebral Infarction (mTICI) grade 2b or 3 or expanded TICI (eTICI) grade 2c/3] following reperfusion therapy without achieving functional independence within 90 days [1-3]. Despite a 71% successful vessel recanalization rate reported by the Highly Effective Reperfusion Evaluated in Multiple Endovascular Stroke Trials (HERMES) collaboration, only 46% of patients exhibited favorable outcomes [4]. Notably, the incidence of futile recanalization is significantly higher in Chinese stroke cohorts, reaching approximately 60% compared to Western populations [5]. As endovascular treatment (EVT) continues to advance and gain widespread adoption, investigating the complex relationship between futile recanalization and patient prognosis has become crucial for enhancing recovery rates and improving the quality of life for patients with acute ischemic stroke (AIS).

The exact mechanisms underlying futile recanalization remain elusive but are believed to involve multiple factors, such as no-reflow phenomena and poor arterial collateral circulation, and so on [6-8]. Existing research has identified multifaceted factors influencing futile recanalization, including patient characteristics (age, sex), National Institutes of Health Stroke Scale [NIHSS] score, laboratory indicators (stress hyperglycemia, brain natriuretic peptide [BNP] and international normalized ratio [INR]), imaging parameters (arterial collateral circulation status and Alberta Stroke Program Early CT Score [ASPECTS]), and procedural factors (time from onset to hospital presentation and number of thrombectomy attempts) [1, 2, 4, 6-8].

However, previous studies have primarily focused on arterial recanalization in cases of cerebral artery stenosis or occlusion, while largely neglecting the “outflow” factors such as venous drainage, cerebrospinal fluid circulation, and the dynamic changes in cerebral tissue perfusion. Recent research has highlighted the importance of a unique circulatory system comprising arteries, veins, glial cells, and the autonomic nervous system in maintaining cerebral homeostasis [9]. This insight suggests that exploring the mechanisms of futile recanalization should consider the overall dynamic coordination from arterial inflow, microvascular and microcirculatory function, to downstream venous drainage. Impaired venous drainage following stroke can lead to elevated venous pressure, potentially influencing patient prognosis through various mechanisms. Although some studies have investigated the relationship between venous drainage and outcomes in AIS patients [10, 11], the specific association between venous drainage and futile recanalization remains unclear. Analyzing the mechanisms of futile recanalization from an integrated arterial-venous perspective may better align with the underlying pathophysiology of the disease and contribute to a more comprehensive understanding of its influencing factors.

In light of this, this multicenter study aims to investigate the occurrence and predictors of futile recanalization following EVT in acute large vessel occlusion stroke, emphasizing the role of arterial-venous collateral circulation. The objectives are:

1. To identify predictors of futile recanalization, focusing on arterial-venous collaterals.
2. To develop and validate a predictive nomogram incorporating arterial-venous collaterals.
3. To provide evidence-based guidance considering arterial-venous collaterals’ impact on outcomes.

## MATERIALS AND METHODS

### Study population

This study consecutively enrolled patients with acute anterior circulation large vessel occlusion who underwent EVT at two centers between March 2016 and June 2024 (Center A: Beijing Hospital; Center B: Xuanwu Hospital, Capital Medical University).

Inclusion criteria were: (1) age ≥ 18 years; (2) time from symptom onset to admission ≤ 24 hours; (3) unilateral internal carotid artery (ICA) or middle cerebral artery (MCA) M1 or M2 segment occlusion; (4) NIHSS score assessed at admission; (5) multimodal CT imaging at admission, including non-contrast CT (NCCT), 4D CTA, and CT perfusion (CTP), with follow-up NCCT or magnetic resonance imaging (MRI) 2-7 days post-procedure; (6) successful vascular recanalization following EVT.

Exclusion criteria were: (1) bilateral lesions or history of large infarction (> 2/3 MCA territory); (2) poor quality imaging; (3) incomplete imaging or clinical data.

The need for patient consent was waived following the ethical standards of the 1964 Declaration of Helsinki and its later amendments.

### Data collection

1. **Demographic data and medical history collection:** Basic demographic information was collected, including age, sex, and other relevant factors. Additionally, a detailed medical history was obtained, focusing on common stroke risk factors such as hypertension, diabetes, previous stroke, transient ischemic attacks (TIAs), atrial fibrillation, and coronary artery disease.
2. **Clinical evaluation:** Upon admission, an experienced neurologist performed a comprehensive clinical evaluation. The NIHSS score was used to quantify stroke severity, with a total score range of 0 to 42. Ischemic stroke etiology was classified according to the Trial of Org 10172 in Acute Stroke Treatment (TOAST) criteria, including subtypes such as large artery atherosclerosis, cardioembolism, other causes, and undetermined causes [12].
3. **Key time points:** Standardized data collection forms were used to record key time intervals for each patient, including time from symptom onset to hospital arrival, time from arrival to imaging, time from imaging to arterial puncture, and time from puncture to recanalization.
4. **Imaging data collection and evaluation:**
  - Assessment of cerebral venous collateral circulation: The venous collateral circulation was evaluated using the venous collateral scale based on 4D CTA. The assessment focused on the opacification of the main cortical veins, including the superficial middle cerebral vein, sphenoparietal sinus, Labbé vein, and Trolard vein. Each vein was scored separately, with a total score range from 0 to 16, where higher scores indicate better venous collateral circulation [13].
  - Assessment of cerebral arterial collateral circulation: The arterial collateral circulation was evaluated using the arterial collateral scale based on 4D CTA, with a total score range of 0 to 4. Higher scores indicate better development of arterial collateral circulation [14].
  - Assessment of ischemia severity: ASPECTS was used on the initial NCCT to perform a rapid, semi-quantitative evaluation of the ischemic extent in the brain.
  - Evaluation of infarct core and ischemic penumbra: Post-processing of the admission CTP images was performed to obtain cerebral blood flow (CBF), cerebral blood volume (CBV), and time to peak (TTP) maps. Infarct core was defined by a reduction in CBV of 38% and a TTP extension > 5.3s, while ischemic penumbra was defined by a TTP extension > 5.3s but without significant CBV reduction. The infarct core volume, ischemic penumbra volume, and mismatch ratio were calculated based on these criteria [15].
  - Thrombus load assessment: The clot burden score (CBS) was used to quantitatively assess thrombus load in the intracranial anterior circulation using admission CTA images, with a total score range from 0 to 10 [16].
5. **Endovascular treatment and outcome evaluation**
  - EVT procedure: Data collected included intra-arterial thrombolysis, stent retriever (SR) thrombectomy, contact aspiration (CA), combined SR and CA (Solumbra technique), and/or percutaneous transluminal angioplasty (PTA) and/or stent placement [13].
  - Clinical outcome evaluation: The modified Rankin Scale (mRS) scores at 90 days post-procedure were recorded by experienced neurologists or neurosurgeons, with mRS 0-2 defined as good outcome [2].
  - Recanalization assessment: The mTICI grading system was used with scores ≥ 2b considered successful. Futile recanalization was defined as poor outcome (mRS > 2) despite successful recanalization [2].
6. **Inter-rater reliability analysis for venous collaterals:** To assess the reliability of our venous collateral scoring system, two experienced neuroradiologists independently evaluated the venous collateral circulation. Given the continuous nature of our scoring system (0-16 points), we employed the Intraclass Correlation Coefficient (ICC) using a two-way random-effects model with absolute agreement.

### Feature analysis, selection, and model construction

1. Dataset splitting and baseline feature comparison: Patients were randomly divided into training and internal validation sets (7:3 ratio) using a random number table. To rigorously assess model generalizability, we utilized patients from Center B as a completely independent external validation cohort. Baseline characteristics were compared between groups to verify balanced data distribution.
2. Feature selection and model construction: Feature selection employed a two-step approach combining Boruta algorithm and LASSO regression to reduce noise in high-dimensional data. Boruta initially identified potential predictors in the training set by comparing original features against random “shadow features”, followed by LASSO regression for further dimensionality reduction.
  Model construction utilized five supervised machine learning algorithms: logistic regression (LR), random forest (RF), support vector machine (SVM), XGBoost, and LightGBM. To mitigate overfitting, we implemented various regularization strategies tailored to each algorithm. For tree-based methods (Random Forest, XGBoost, LightGBM), we utilized pruning techniques, optimized minimum samples per leaf, and controlled tree depth to prevent overfitting. For logistic regression, we employed L2 regularization with cross-validation for parameter optimization. SVM regularization was achieved through optimal C parameter selection balancing model complexity and performance. Detailed parameter configurations are provided in Supplementary Table 1. Hyperparameter tuning with 5-fold cross-validation was implemented to optimize performance and minimize overfitting.
3. Subgroup Sensitivity Analysis: To evaluate model robustness and transportability across heterogeneous patient populations, we performed pre-specified subgroup analyses stratified by clinically relevant baseline characteristics. Patients were stratified by age (≤65 vs >65 years), atrial fibrillation status (yes or no), baseline NIHSS severity (≤15 vs >15, with NIHSS >15 indicating severe neurological deficit according to established clinical criteria), and prior stroke/TIA history (yes or no). Model performance was evaluated within each subgroup using identical performance metrics as the primary analysis.
4. Model assessment and interoretation: Model performance was assessed by discrimination (area under the receiver operating characteristic curve [AUC]), calibration (Hosmer-Lemeshow test, calibration curves), and clinical utility (decision curve analysis [DCA]).
  For interpretability, Shapley Additive Explanation (SHAP) values were used to rank feature contributions based on their marginal impact on model outputs. Additionally, local Interpretable Model-agnostic Explanations (LIME) were employed to generate instance-specific explanations, enhancing transparency for clinical stakeholders.
5. Clinical application and development of predictive tools: An interactive web-based calculator was developed to facilitate real-time predictions of futile recanalization risk based on individualized patient parameters, supporting clinical decision-making.

### Statistical analysis

All data analyses were performed using R software (version 4.2.2) and Python (version 3.4.3). Continuous variables were presented as mean ± standard deviation or median (interquartile range) and compared via t-tests or Mann-Whitney U tests. Categorical variables were expressed as frequency (percentage) and compared using χ² or Fisher’s exact tests. Missing data were addressed through multiple imputations (mice package v3.14.0).

For feature selection, the “Boruta” package (ntree = 500, maxRuns = 500, *P* < 0.01) initially screened potential predictors in the training set. Subsequently, LASSO regression (“glmnet” package) with optimal λ determined by 10-fold cross-validation further reduced model dimensionality.

Model performance was assessed using AUC (discrimination), Hosmer-Lemeshow test and calibration curves (calibration), and decision curve analysis via “rmda” package (clinical utility). All tests were two-tailed with statistical significance at *P* < 0.05.

**Figure 1.**
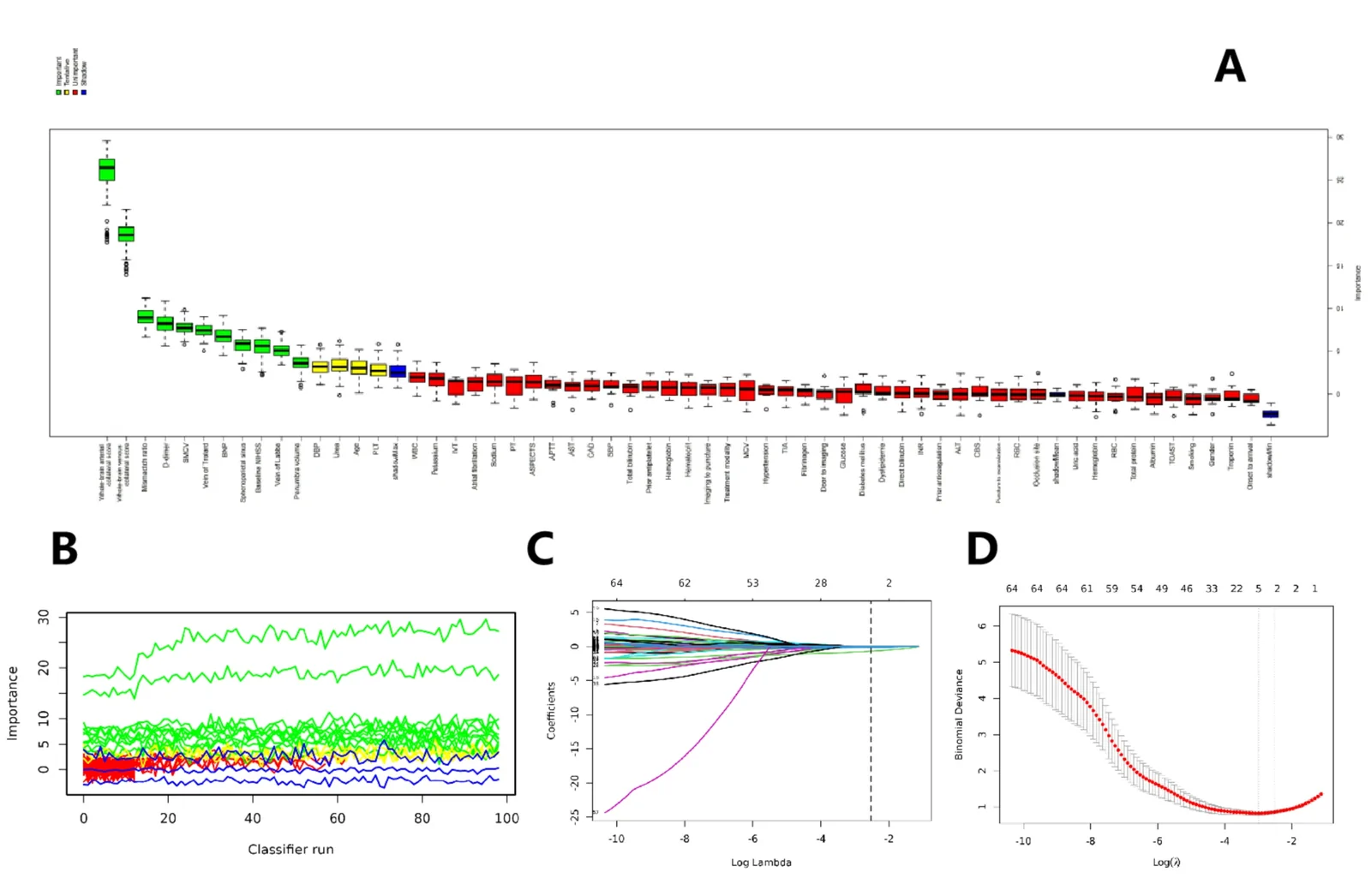
**Features selected by boruta and lasso analysis**. (**A**) Variables selected by Boruta algorithm (training cohort, n=160). The minimum, average and maximum shadow score are shown in blue. Based on feature importance scores, 11 variables marked in green are identified as important variables, while yellow variables are considered neutral and red variables are rejected. (**B**) The importance scores from Boruta algorithm across 100 classifier runs, showing the stability and consistency of variable selection over multiple iterations. (**C**) The Lasso regression coefficient profiles showing how different baseline characteristics’ coefficients change with varying levels of regularization. (**D**) The optimal lambda selection in Lasso regression using 10-fold cross-validation. The plot shows binomial deviance against log(lambda), with error bars representing cross-validation error estimates.

## RESULTS

### Baseline Characteristics of Study Cohorts

This study enrolled 392 subjects who were stratified into three cohorts: training (n = 160) and internal validation (n = 69) from Center A, and external validation (n = 163) exclusively from Center B (Supplementary Fig. 1). Median age was 68 years overall (interquartile range [IQR]: 59-78), with cohort-specific medians of 73 years in both training (IQR: 63-82) and internal validation (IQR: 61-81) cohorts, and 64 years (IQR: 55-70) in the external validation cohort. Males constituted 67.9% of participants overall (63.1%, 58.0%, and 76.7% in training, internal validation, and external validation cohorts, respectively).

Significant inter-cohort differences in cardiovascular risk factors were observed. For example, the external validation cohort exhibited the highest prevalence of atrial fibrillation (80.4%, *P* < 0.001) and hypertension (58.3%, *P* = 0.005). Nonetheless, baseline characteristics and clinical outcomes were statistically comparable across all three cohorts (Supplementary Table 2 and Supplementary Table 3).

Inter-rater reliability analysis for the venous collateral scoring system demonstrated excellent agreement between evaluators with an ICC of 0.920 (95% CI: 0.904-0.934).

### Feature Selection and Independent Risk Factors for Recanalization Failure

The feature selection process consisted of two steps: the Boruta algorithm followed by LASSO regression (Fig. 1). The Boruta algorithm, utilizing random forest modeling, identified 11 predictive variables: BNP, D-dimer levels, whole-brain arterial collateral circulation status, whole-brain venous collateral circulation status, vein of Trolard, vein of Labbé, sphenoparietal sinus, superficial middle cerebral vein, perfusion mismatch ratio, penumbra volume, and admission NIHSS scores.

LASSO regression analysis, with cross-validation (optimal λ value = 0.0798), subsequently identified three primary predictors: age, whole-brain arterial collateral circulation status, and whole-brain venous collateral circulation status.

### Evaluation of Different Model

In the training cohort, XGBoost demonstrated optimal performance (AUC: 0.988, 95% CI: 0.981-0.996; F1: 0.935, 95% CI: 0.926-0.944; accuracy: 0.942, 95% CI: 0.933-0.952; kappa: 0.883, 95% CI: 0.864-0.901), followed by LightGBM (AUC: 0.967, 95% CI: 0.951-0.983; F1: 0.894, 95% CI: 0.883-0.906), Logistic Regression (AUC: 0.862, 95% CI: 0.819-0.904; F1: 0.816, 95% CI: 0.810-0.821), Random Forest (AUC: 0.850, 95% CI: 0.807-0.894; F1: 0.767, 95% CI: 0.752-0.783), and SVM (AUC: 0.832, 95% CI: 0.787-0.877; F1: 0.762, 95% CI: 0.753-0.771) (Supplementary Fig. 2).

In the test cohort, Logistic Regression achieved the highest performance (AUC: 0.859, 95% CI: 0.773-0.945; F1: 0.806, 95% CI: 0.779-0.833; accuracy: 0.828, 95% CI: 0.806-0.849; kappa: 0.651, 95% CI: 0.607-0.696), followed by Random Forest (AUC: 0.850, 95% CI: 0.763-0.936; F1: 0.735, 95% CI: 0.693-0.776), LightGBM (AUC: 0.845, 95% CI: 0.756-0.935; F1: 0.764, 95% CI: 0.715-0.812), XGBoost (AUC: 0.838, 95% CI: 0.745-0.930; F1: 0.764, 95% CI: 0.714-0.814), and SVM (AUC: 0.831, 95% CI: 0.741-0.921; F1: 0.743, 95% CI: 0.721-0.765) (Supplementary Table 4 and Supplementary Table 5).

The performance discrepancy between training and test cohorts suggests that Logistic regression maintained consistent performance across datasets, demonstrating superior generalizability and enhanced suitability for clinical implementation.

### Subgroup Sensitivity Analysis

To assess model transportability, we performed subgroup analyses across key patient characteristics (Supplementary Tables 6 and 7). The logistic regression model demonstrated the most stable performance across subgroups with minimal overfitting (mean training-test ΔAUC = 0.138), followed by LightGBM (ΔAUC = 0.155) and XGBoost (ΔAUC = 0.194). Specifically, the logistic regression model maintained robust performance across age groups (≤65 years: training AUC=0.883, external validation AUC=0.739; >65 years: training AUC=0.883, external validation AUC=0.812), atrial fibrillation status (AF-negative: training AUC=0.911, external validation AUC=0.834; AF-positive: training AUC=0.863, external validation AUC=0.533), and NIHSS severity (≤15: training AUC=0.865, external validation AUC=0.778; >15: training AUC=0.951, external validation AUC=0.757). Notably, the external validation cohort included no patients with prior stroke/TIA, limiting subgroup analysis to TIA-negative patients where the logistic regression model showed good performance (training AUC=0.904, external validation AUC=0.754). Across all evaluable subgroups, logistic regression consistently ranked first or second among all algorithms tested, demonstrating superior stability and generalizability despite significant inter-cohort baseline differences.

**Table 1:** Univariate and Multivariate Logistic Regression Analysis.

Characteristic	Univariate OR (95% CI)	*P*-value	Multivariate OR (95% CI)	*P*-value
**Age**	1.07 (1.04, 1.10)	< 0.001	1.06 (1.02, 1.11)	0.003
**Whole-brain arterial collateral score**	0.23 (0.14, 0.36)	< 0.001	0.30 (0.18, 0.50)	< 0.001
**Whole-brain venous collateral score**	0.64 (0.56, 0.74)	< 0.001	0.78 (0.67, 0.90)	< 0.001

OR = Odds Ratio, CI = Confidence Interval

### Univariate and Multivariate Analysis

Univariate analysis of the training cohort (N = 160, recanalization failure events = 63) identified significant associations between recanalization failure and three variables. Age demonstrated a positive association with recanalization failure (odds ratio [OR]: 1.07; 95% confidence interval [CI]: 1.04-1.10; *P* < 0.001), indicating that each year increase in age was associated with a 7% higher risk of failure. The whole-brain arterial collateral circulation status showed a protective effect (OR: 0.23; 95% CI: 0.14-0.36; *P* < 0.001), suggesting that better arterial collateral circulation was associated with a 77% lower risk of recanalization failure. Similarly, better whole-brain venous collateral circulation demonstrated a protective effect (OR: 0.64; 95% CI: 0.56-0.74; *P* < 0.001), indicating a 36% decrease in futile recanalization risk (Table 1).

In multivariate logistic regression analysis, all three variables remained significant independent predictors. After adjustment for other variables, age maintained its association with increased risk (OR: 1.06; 95% CI: 1.02-1.11; *P* = 0.003). The whole-brain arterial collateral circulation status remained protective (OR: 0.30; 95% CI: 0.18-0.50; *P* < 0.001), as did the whole-brain venous collateral circulation status (OR: 0.78; 95% CI: 0.67-0.90; *P* < 0.001) (Table 1).

ROC analysis demonstrated strong discriminative ability for the collateral circulation measures. Age demonstrated moderate discriminative performance (AUC: 0.711; 95% CI: 0.629-0.793). The whole-brain arterial collateral circulation status achieved an AUC of 0.848 (95% CI: 0.782-0.913), while the whole-brain venous collateral circulation status showed an AUC of 0.853 (95% CI: 0.788-0.918). (Supplementary Table 8).

**Figure 2.**
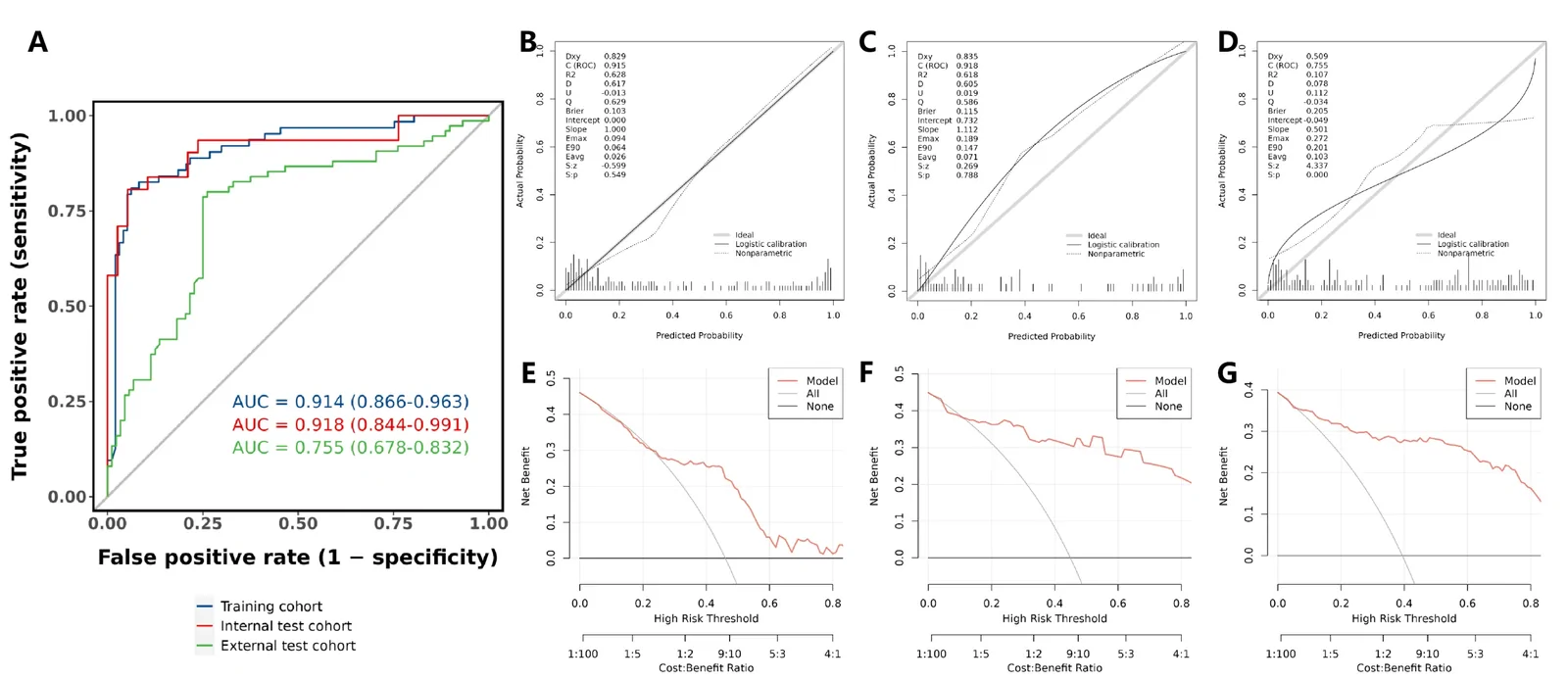
**Comprehensive evaluation of the predictive model across different cohorts**. (**A**) Receiver operating characteristic (ROC) curves illustrating the discrimination performance of the model in the training cohort (blue line; n=160: effective=97, futile=63), internal test cohort (red line; n=69: effective=38, futile=31), and external test cohort (green line; n=163: effective=88, futile=75). The area under the curve (AUC) values with 95% confidence intervals are provided for each cohort, calculated using DeLong's method. (**B-D**) Calibration plots assessing the agreement between predicted probabilities and observed outcomes in the training cohort (B), internal test cohort (C), and external test cohort (D). Calibration was evaluated using the Hosmer-Lemeshow test with Brier scores of 0.103, 0.115, and 0.205, respectively. (**E-G**) Decision curve analysis (DCA) for the training cohort (E), internal test cohort (F), and external test cohort (G), demonstrating net benefit across threshold probabilities of 0.2-0.6, with the model providing superior clinical utility compared to "treat all" or "treat none" strategies.

**Figure 3.**
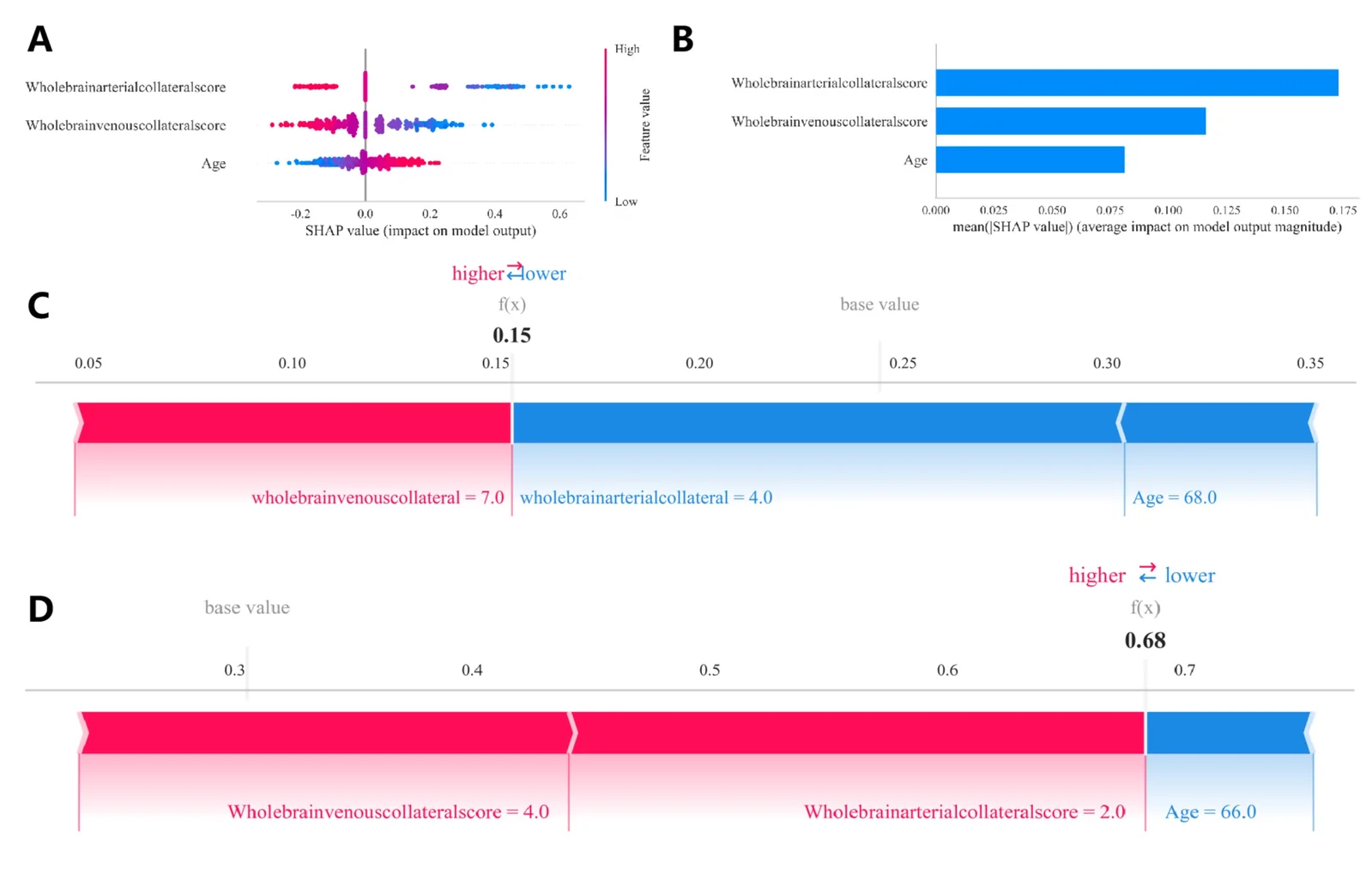
**Interpretability analysis of logistic regression models**. (**A**) SHAP (SHapley Additive exPlanations) summary plot showing the impact of each feature on model output across all samples in the training cohort (n=160). Each point represents a sample, with color indicating feature value (red=high, blue=low) and horizontal position showing SHAP value (impact on model output). (**B**) Feature importance ranking based on mean absolute SHAP values, quantifying the average impact magnitude of each feature on model predictions. (**C-D**) LIME (Local Interpretable Model-agnostic Explanations) analysis for two representative cases from the validation cohort: (C) a case with low predicted probability of futile recanalization (f(x)=0.15), and (D) a case with high predicted probability of futile recanalization (f(x)=0.68), showing how individual feature values contribute to the final prediction.

### Model Performance and Clinical Implementation

The logistic regression model demonstrated consistent discriminative performance across all cohorts (Fig. 2A). In the training cohort, the model achieved an AUC of 0.914 (95% CI: 0.866-0.963), with comparable results in the internal validation cohort (AUC: 0.918; 95% CI: 0.844-0.991). Generalizability was further validated in the external cohort (AUC: 0.755; 95% CI: 0.678-0.832), with confidence intervals estimated via DeLong’s method.

Calibration analysis indicated close alignment between predicted and observed outcomes (Fig. 2B-D). The training cohort exhibited near-ideal calibration (Brier score: 0.103), the internal validation cohort showed minor deviations (Brier score: 0.115), and the external cohort maintained stable calibration despite clinical heterogeneity (Brier score: 0.205).

Decision curve analysis (Fig. 2E-G) demonstrated clinical utility across threshold probabilities of 0.2-0.6, with the model providing higher net benefit than “treat all” or “treat none” strategies, supporting its utility for clinical decision-making.

SHAP analysis highlighted whole brain arterial collateral score as the primary predictor, followed by venous collateral score and age. Case-level analysis illustrated these features’ influence on individual predictions (Fig. 3).

**Figure 4.**
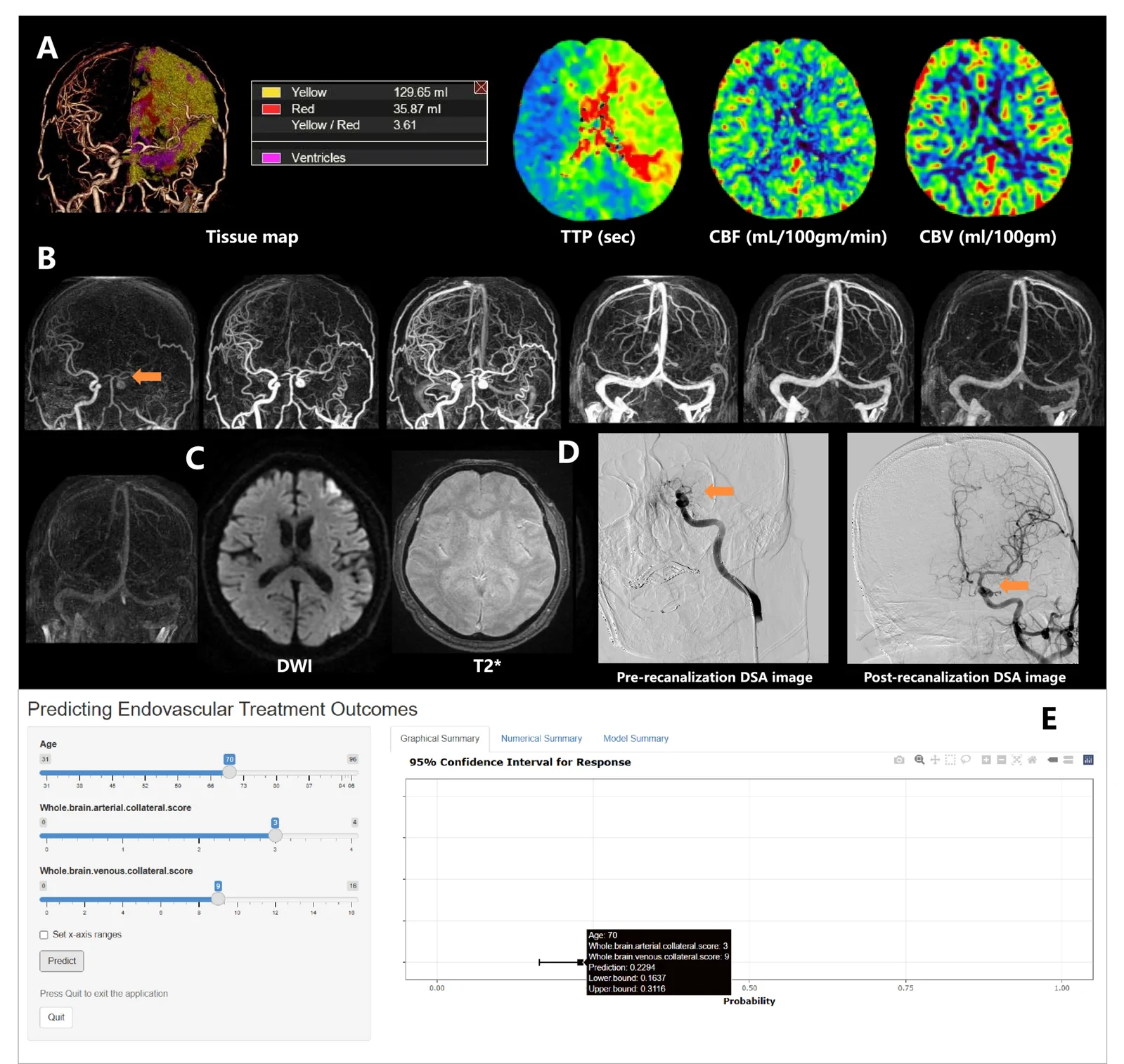
**Case of effective recanalization and probability estimation**. A 70-year-old patient presented with dizziness lasting 3 hours. Medical history included hypertension and liver disease. Upon admission, the NIHSS score was 1. (**A-B**) CTA revealed left internal carotid artery occlusion, and CTP showed prolonged TTP in the left MCA territory, with mildly reduced CBF and no significant change in CBV. The whole-brain collateral scores were 3 for arterial and 9 for venous. (**C-D**) After EVT, the mTICI score was 3. At 90-day follow-up, the mRS score was 0, indicating a favorable outcome and effective recanalization. (**E**) The dynamic prediction system assessed the risk of ineffective recanalization for this patient. The clinical parameters entered were: age 70, whole-brain arterial collateral score of 3, and venous collateral score of 9. The system predicted a 22.94% probability of ineffective recanalization, consistent with the favorable outcome.

We developed an interactive web-based calculator (https://predicting.shinyapps.io/dynnomapp/) for real-time risk estimation. Representative cases of effective (Fig. 4) and futile recanalization (Fig, 5) illustrate the tool’s integration into clinical assessment workflows.

## DISCUSSION

The present investigation established and validated a prognostic nomogram for predicting futile recanalization following EVT in patients with acute ischemic stroke. This novel model incorporates critical hemodynamic parameters, specifically the whole-brain arterial collateral circulation status and whole-brain venous collateral circulation status. The model exhibited excellent discriminatory ability across multiple cohorts: the training cohort (AUC = 0.914), internal validation cohort (AUC = 0.918), and completely independent external validation cohort (AUC = 0.755). Furthermore, calibration analyses indicated good agreement between predicted and observed outcomes. Decision curve analysis further suggested that applying this model for clinical decision-making within a specific threshold range yielded net benefits. These findings indicate that a predictive model incorporating both arterial and venous assessments may provide valuable insights for clinical practice.

Notably, this study introduced the innovative concept of “whole-brain arterial-venous collateral circulation” to enhance the comprehensive evaluation of overall cerebrovascular status in AIS patients. The intracranial venous system, comprising approximately 70% of total cerebral blood volume, has traditionally been considered merely a passive component in cerebrovascular circulation [17, 18]. However, accumulating evidence has revealed its integral function in maintaining cerebral perfusion pressure and regulating regional hemodynamic parameters [11, 13, 19-22]. This advanced understanding of venous circulation highlights the importance of incorporating venous system evaluation into clinical imaging protocols, which may provide new insights into AIS pathophysiology and treatment strategies.

**Figure 5.**
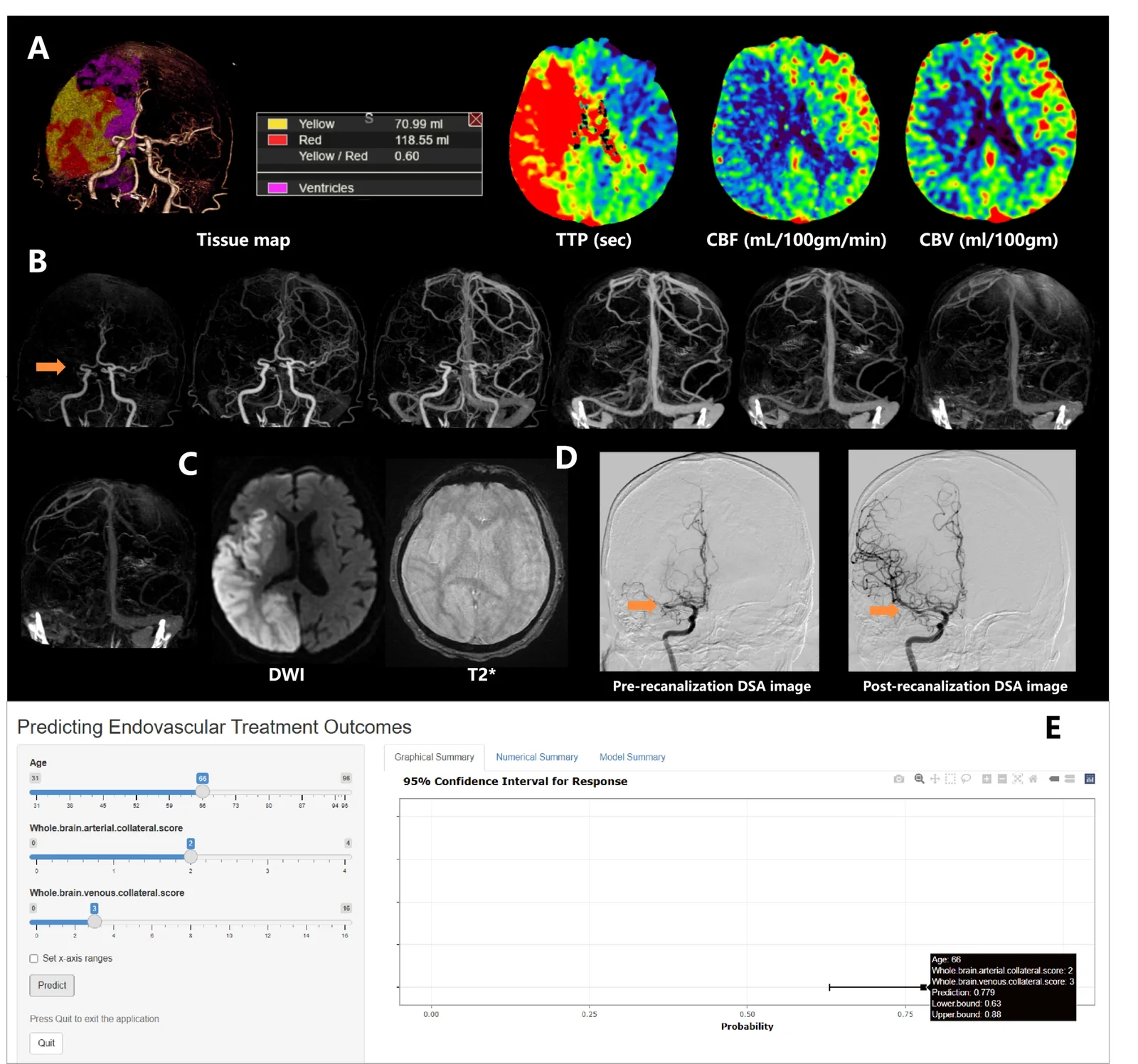
**Case of futile recanalization and probability estimation**. A 66-year-old patient presented with left-sided limb weakness and slurred speech for 8 hours. Medical history included coronary artery disease. The NIHSS score was 4. (**A-B**) CTA revealed right MCA occlusion, and CTP showed prolonged TTP with reduced CBF and localized CBV reduction. The whole-brain collateral scores were 2 for arterial and 3 for venous. (**C-D**) After EVT, the mTICI score was 3. At 90-day follow-up, the mRS score was 4, indicating an unfavorable outcome and futile recanalization. (**E**) The dynamic prediction system assessed the risk of futile recanalization for this patient. Clinical parameters entered were: age 66, whole-brain arterial collateral score of 2, and venous collateral score of 3. The system predicted a 77.90% probability of futile recanalization, consistent with the poor outcome.

The mechanistic significance of venous circulation in AIS pathophysiology transcends its volumetric predominance in cerebral vasculature. While arterial collateral circulation has been extensively documented as a compensatory mechanism in ischemic territories, mounting evidence suggests that venous drainage status may constitute a more sensitive indicator of tissue perfusion quality [11, 13, 21]. Recent investigations have delineated several critical protective mechanisms through which efficient venous drainage influences stroke outcomes: (1) Adequate venous drainage helps maintain optimal blood flow velocity, reducing the likelihood of thrombus formation [23]; (2) effective venous outflow accelerates the clearance of metabolic waste products in ischemic areas, improving the microenvironment [24]; (3) the venous system can compensate through collateral network remodeling [25]. These potential mechanisms highlight the crucial role of the venous system in the development and progression of ischemic stroke. Therefore, when venous system function is impaired, even if arterial recanalization is achieved, local tissue perfusion may remain suboptimal. This observation underscores the importance of evaluating not only arterial patency but also the functional status of the venous system when assessing the efficacy of vascular recanalization.

Based on this understanding, we evaluated venous collateral circulation and integrated it with arterial collateral circulation scores to comprehensively assess cerebrovascular hemodynamics. Our approach aims to provide a more holistic representation of cerebral hemodynamic changes, strengthening support for clinical decision-making. For example, during the treatment decision-making process, identifying high-risk patients could guide the implementation of apposite interventions or closer monitoring, ultimately improving outcomes.

Additionally, this study utilized statistical methods such as Boruta and LASSO regression to identify predictive factors, ultimately determining venous collateral circulation score, arterial collateral circulation score, and age as the three main predictive indicators. The selection of these indicators was based on their clinical accessibility and the reliability of their predictive performance. The model development process employed rigorous statistical methodologies, including cross-validation and testing in independent validation cohorts, which enhanced the model's stability and generalizability. In clinical application, this model has the potential to assist physicians in evaluating patients’ prognostic risk prior to treatment, providing a foundation for developing individualized therapeutic strategies. Moreover, it may guide the optimization of monitoring strategies during treatment for high-risk patients. The development of an online prediction tool further simplifies the clinical application of the model, enabling physicians to quickly assess patient risk. However, the effectiveness of this tool requires further validation through extensive clinical practice.

Our predictive model demonstrated excellent discriminative ability across all cohorts (AUC: 0.914 for training, 0.918 for internal validation, and 0.755 for external validation); however, calibration performance varied with Brier scores of 0.103, 0.115, and 0.205, respectively. Patient characteristics differed substantially between centers: the external cohort had a younger population (median age 64 versus 73 years), lower atrial fibrillation prevalence (19.6% versus 39.4%), and higher baseline stroke severity (median NIHSS 16.0 versus 12.0), which altered the underlying risk distribution and affected probability estimates. Technical variations in imaging protocols also contributed to calibration differences, including differences in contrast injection protocols and image reconstruction parameters between centers, which may have affected collateral visualization quality despite good inter-observer agreement (ICC=0.92). Future studies should standardize imaging acquisition protocols to improve model transferability across centers while maintaining robust discriminative performance.

Despite the significant findings of this study, several limitations should be acknowledged. First, as a retrospective study, it is challenging to completely avoid selection bias and information bias, particularly during data collection. Incomplete records may have led to the omission of some key information, potentially impacting the accuracy of the results. Second, although the model's discriminative performance was validated using an independent external cohort, the observed calibration differences (Brier score: 0.205 versus 0.103-0.115 in internal cohorts) highlighted the challenges of cross-institutional model implementation. These calibration discrepancies likely reflected patient population heterogeneity and center-specific variations in imaging protocols and collateral assessment techniques. Additionally, the assessment of venous collateral circulation currently lacks standardized criteria, with variations in evaluation methods across centers affecting result comparability. Third, while we collected glucose parameters in our study, they were not retained as significant predictors during feature selection, possibly due to our focus on comprehensive vascular imaging parameters. Future studies should explore the integration of metabolic factors such as stress hyperglycemia indices with detailed vascular assessments to develop more comprehensive predictive models. The integration of metabolic biomarkers with vascular assessment could potentially enhance predictive accuracy. These efforts will enhance reproducibility and optimize the clinical implementation of this integrated assessment approach, ultimately improving EVT decision-making in acute ischemic stroke patients.

In conclusion, this study emphasizes the importance of integrating arterial and venous collateral circulation assessment in AIS prognosis prediction. Our predictive model demonstrated robust discrimination and calibration capabilities through both internal and external validations, highlighting the clinical viability of this integrated approach. Implementation of these findings requires three critical developments: large-scale multicenter validation studies, standardization of venous collateral circulation assessment protocols, and elucidation of underlying pathophysiological mechanisms. These advancements will optimize AIS treatment strategies and provide a more comprehensive framework for evidence-based clinical decision-making, so that reduce the possibility of ineffective recanalization.

## Supplementary Materials

The Supplementary data can be found online at: www.aginganddisease.org/EN/10.14336/AD.2025.0540.

## Data Availability

Data will be made available on request.
