# Supplementary material for: Dynamic CTA-Based Whole-Brain Arterial-Venous Collateral Assessment for Predicting Futile Recanalization in Acute Ischemic Stroke
Source: Aging Dis. 2025 Jun 8;17(4):2154–65. doi: 10.14336/AD.2025.0540 (PMC13256342; doi:10.14336/AD.2025.0540)
Supplement: Supplementary file 1 — The Supplementary data can be found online at: www.aginganddisease.org/EN/10.14336/AD.2025.0540. [file AD-17-4-2154-s.pdf]

SUPPLEMENTARY DATA

Dynamic CTA-Based Whole-Brain Arterial-Venous Collateral Assessment for Predicting Futile Recanalization in Acute Ischemic Stroke

Ruoyao Cao, Yao Lu, Wei Li, Fan Yu, Shen Hu, Kunpeng Chen, Guoxuan Wang, Chengkan Sun, Qingfeng Ma, Miao Zhang, Juan Chen, Jie Lu

© 2025. Cao R et al. Published online at http://www.aginganddisease.org/EN/10.14336/AD.2025.0540

SUPPLEMENTARY DATA

Supplementary Table 1. Machine Learning Model Hyperparameters.

Model	Key Parameters
**XGBClassifier**	• objective='binary:logistic' • learning_rate=0.3 • max_depth=6 • min_child_weight=3 • reg_lambda=1.0
**Logistic Regression**	• C=1.0 • penalty='L2' • max_iter=100 • tol=0.0001
**Random Forest**	• criterion='gini' • max_depth=10 • min_impurity_decrease=0.1 • n_estimators=100
**SVM**	• C=1.0 • kernel='rbf' • tol=0.1
**LightGBM**	• boosting_type='gbdt' • learning_rate=0.3 • max_depth=6 • num_leaves=31 • n_estimators=100

Supplementary Table 2. Patient demographics and baseline characteristics.

Characteristic	Cohort				P-value[2]
	Overall, N = 392[1]	Training Cohort, N = 160[1]	Internal Test Cohort, N = 69[1]	External Test Cohort, N = 163[1]	
**Demographic characteristic**					
Age, median (IQR), years	68 (59, 78)	73 (63, 82)	73 (61, 81)	64 (55, 70)	<0.001
Sex, n (%)					0.005
Female	126 (32.1%)	59 (36.9%)	29 (42.0%)	38 (23.3%)	
Male	266 (67.9%)	101 (63.1%)	40 (58.0%)	125 (76.7%)	
**Intravenous thrombolysis, n (%)**					0.278
No	287 (73.2%)	124 (77.5%)	49 (71.0%)	114 (69.9%)	
Yes	105 (26.8%)	36 (22.5%)	20 (29.0%)	49 (30.1%)	
**Baseline NIHSS, median (IQR)**	13.0 (9.0, 18.0)	12.0 (8.0, 16.0)	11.0 (7.0, 15.0)	16.0 (12.0, 19.0)	<0.001
**Clinical characteristics**					
Atrial fibrillation					<0.001
No	267 (68.1%)	97 (60.6%)	39 (56.5%)	131 (80.4%)	
Yes	125 (31.9%)	63 (39.4%)	30 (43.5%)	32 (19.6%)	
Hypertension					0.005
No	128 (32.7%)	42 (26.3%)	18 (26.1%)	68 (41.7%)	
Yes	264 (67.3%)	118 (73.8%)	51 (73.9%)	95 (58.3%)	
Diabetes mellitus					0.064
No	263 (67.1%)	101 (63.1%)	42 (60.9%)	120 (73.6%)	
Yes	129 (32.9%)	59 (36.9%)	27 (39.1%)	43 (26.4%)	
Dyslipidemia					<0.001
No	249 (63.5%)	93 (58.1%)	29 (42.0%)	127 (77.9%)	
Yes	143 (36.5%)	67 (41.9%)	40 (58.0%)	36 (22.1%)	
Current smoking					0.118
No	254 (64.8%)	110 (68.8%)	48 (69.6%)	96 (58.9%)	
Yes	138 (35.2%)	50 (31.3%)	21 (30.4%)	67 (41.1%)	
Prior stroke/TIA					<0.001
No	301 (76.8%)	95 (59.4%)	43 (62.3%)	163 (100.0%)	
Yes	91 (23.2%)	65 (40.6%)	26 (37.7%)	0 (0.0%)	
Coronary artery disease					<0.001
No	269 (68.6%)	97 (60.6%)	39 (56.5%)	133 (81.6%)	
Yes	123 (31.4%)	63 (39.4%)	30 (43.5%)	30 (18.4%)	
Prior anticoagulation therapy					<0.001
No	355 (90.6%)	134 (83.8%)	58 (84.1%)	163 (100.0%)	
Yes	37 (9.4%)	26 (16.3%)	11 (15.9%)	0 (0.0%)	
Prior antiplatelet therapy					<0.001
No	334 (85.2%)	122 (76.3%)	49 (71.0%)	163 (100.0%)	
Yes	58 (14.8%)	38 (23.8%)	20 (29.0%)	0 (0.0%)	
**Stroke characteristics**					
TOAST classification, n (%)					0.008
Large-artery atherosclerosis	195 (49.7%)	69 (43.1%)	29 (42.0%)	97 (59.5%)	
Cardioembolism	173 (44.1%)	84 (52.5%)	34 (49.3%)	55 (33.7%)	
Other determined etiology	8 (2.0%)	1 (0.6%)	3 (4.3%)	4 (2.5%)	
Undetermined etiology	16 (4.1%)	6 (3.8%)	3 (4.3%)	7 (4.3%)	
Occlusion site, n (%)					0.427
ICA	134 (34.2%)	53 (33.1%)	18 (26.1%)	63 (38.7%)	
M1	180 (45.9%)	68 (42.5%)	37 (53.6%)	75 (46.0%)	
M2	63 (16.1%)	32 (20.0%)	11 (15.9%)	20 (12.3%)	
Tandem	15 (3.8%)	7 (4.4%)	3 (4.3%)	5 (3.1%)	
**Imaging characteristics**					
Clot burden score, median (IQR)	6.00 (4.00, 8.00)	6.00 (4.00, 8.00)	6.00 (4.00, 8.00)	6.00 (4.00, 7.00)	0.108

© 2025. Cao R et al. Published online at http://www.aginganddisease.org/EN/10.14336/AD.2025.0540

SUPPLEMENTARY DATA

Characteristic	Cohort				P-value[2]
	Overall, N = 392[1]	Training Cohort, N = 160[1]	Internal Test Cohort, N = 69[1]	External Test Cohort, N = 163[1]	
Whole-brain arterial collateral score, median (IQR)	2.00 (1.00, 3.00)	3.00 (2.00, 3.00)	3.00 (1.00, 4.00)	2.00 (1.00, 3.00)	<0.001
ASPECTS, median (IQR)	8.00 (6.00, 9.00)	7.00 (6.00, 9.00)	8.00 (6.00, 9.00)	8.00 (7.00, 9.00)	0.035
Penumbra volume, median (IQR), mL	115 (60, 183)	90 (46, 134)	81 (42, 131)	172 (111, 223)	<0.001
Mismactch ratio, median (IQR)	4 (2, 10)	3 (2, 6)	3 (2, 7)	8 (3, 22)	<0.001
Whole-brain venous collateral score, median (IQR)	8.0 (5.0, 10.0)	8.0 (5.0, 10.3)	9.0 (6.0, 10.0)	7.0 (4.0, 9.0)	0.023
Superficial middle cerebral vein, median (IQR)	2.00 (0.00, 3.00)	2.00 (0.00, 3.00)	3.00 (1.00, 3.00)	1.00 (0.00, 3.00)	0.370
Sphenoparietal sinus, median (IQR)	0.00 (0.00, 2.00)	0.00 (0.00, 3.00)	0.00 (0.00, 2.00)	0.00 (0.00, 1.00)	0.028
Vein of Labbé, median (IQR)	2.00 (1.00, 3.00)	2.00 (1.00, 3.00)	3.00 (1.00, 3.00)	2.00 (1.00, 3.00)	0.101
Vein of Trolard, median (IQR)	3.00 (2.00, 3.00)	3.00 (3.00, 3.00)	3.00 (2.00, 3.00)	3.00 (1.00, 3.00)	0.088
**Treatment characteristics**					
Treatment modality, n (%）					<0.001
ASP	90 (23.0%)	28 (17.5%)	13 (18.8%)	49 (30.1%)	
IA-Tpa	20 (5.1%)	10 (6.3%)	10 (14.5%)	0 (0.0%)	
PTAS	88 (22.4%)	40 (25.0%)	19 (27.5%)	29 (17.8%)	
Solumbra	159 (40.6%)	69 (43.1%)	23 (33.3%)	67 (41.1%)	
SR	31 (7.9%)	13 (8.1%)	4 (5.8%)	14 (8.6%)	
SWIM	4 (1.0%)	0 (0.0%)	0 (0.0%)	4 (2.5%)	
Time metrics, median (IQR), min					
Onset to arrival	251 (112, 520)	214 (100, 529)	204 (91, 320)	321 (171, 541)	0.009
Door to imaging	40 (26, 60)	47 (27, 63)	47 (29, 69)	32 (23, 48)	<0.001
Imaging to puncture	117 (77, 166)	86 (63, 115)	79 (57, 109)	169 (139, 219)	<0.001
Puncture to recanalization	102 (64, 153)	85 (59, 136)	84 (52, 107)	134 (94, 168)	<0.001
**Laboratory parameters**					
Blood pressure, median (IQR), mmHg					
Diastolic	80 (72, 90)	80 (71, 90)	80 (72, 89)	82 (72, 91)	0.566
Systolic	144 (130, 160)	144 (132, 158)	144 (135, 162)	144 (128, 160)	0.800
Blood biochemistry, median (IQR)					
Glucose, mmol/L	7.2 (5.9, 8.7)	7.1 (6.0, 8.6)	7.8 (6.5, 10.9)	6.9 (5.6, 8.2)	0.006
Total protein, g/L	65 (60, 70)	64 (60, 70)	68 (61, 73)	65 (60, 69)	0.138
Albumin, g/L	40.0 (36.0, 43.0)	40.0 (35.0, 44.0)	41.0 (36.0, 44.0)	39.7 (37.6, 41.6)	0.363
ALT, U/L	16 (12, 24)	14 (10, 21)	14 (11, 19)	20 (15, 30)	<0.001
AST, U/L	20 (14, 26)	18 (14, 23)	18 (14, 21)	24 (18, 30)	<0.001
Total bilirubin, μmol/L	13 (9, 17)	13 (10, 18)	12 (9, 15)	13 (9, 18)	0.071
Direct bilirubin, μ mol/L	4.7 (3.2, 6.9)	5.2 (3.7, 7.1)	4.2 (3.3, 6.1)	4.4 (2.8, 6.9)	0.030
Creatinine, μmol/L	78 (65, 99)	75 (65, 88)	71 (62, 86)	85 (65, 109)	0.011
Urea, mmol/L	8 (5, 285)	5 (4, 7)	5 (4, 7)	314 (253, 384)	<0.001
Uric acid, μmol/L	305 (244, 368)	338 (266, 411)	300 (238, 362)	284 (230, 342)	<0.001
Sodium, mmol/L	140.50 (138.50, 142.60)	140.60 (138.58, 142.60)	139.60 (137.60, 141.90)	140.80 (138.85, 142.60)	0.113
Potassium, mmol/L	3.90 (3.68, 4.20)	3.90 (3.68, 4.20)	4.00 (3.70, 4.30)	3.90 (3.60, 4.20)	0.376
Coagulation profile, median (IQR)					
D-dimer, μg/L	253 (139, 594)	273 (148, 617)	238 (103, 548)	252 (139, 591)	0.600
PT, s	11.40 (10.50, 12.10)	11.28 (10.60, 12.10)	11.50 (10.70, 12.20)	11.60 (10.45, 12.05)	0.825
APTT, s	32.1 (29.5, 35.0)	32.6 (30.0, 35.4)	31.7 (29.5, 34.0)	32.5 (29.3, 35.3)	0.273
Fibrinogen, g/L	3.05 (2.60, 3.56)	3.09 (2.69, 3.56)	2.96 (2.58, 3.72)	2.98 (2.53, 3.52)	0.353
INR	0.98 (0.91, 1.03)	0.97 (0.91, 1.03)	0.99 (0.91, 1.05)	0.98 (0.91, 1.04)	0.327
Complete blood count, median (IQR)					
RBC count, $\times 10^{12}$/L	4.35 (3.90, 4.94)	4.28 (3.86, 4.83)	4.41 (3.92, 4.86)	4.41 (4.04, 5.14)	0.183
Platelet count, $\times 10^{9}$/L	202 (169, 244)	198 (169, 229)	195 (157, 229)	209 (175, 250)	0.072
WBC count, $\times 10^{9}$/L	8.26 (6.58, 10.42)	8.10 (6.37, 9.54)	7.96 (6.16, 10.99)	8.62 (6.82, 10.62)	0.165
Hemoglobin, g/L	133 (119, 149)	134 (120, 148)	133 (120, 152)	132 (119, 150)	0.942
Hematocrit, %	40.3 (36.8, 43.9)	38.7 (35.2, 43.4)	39.0 (35.9, 43.1)	41.8 (38.0, 45.0)	<0.001
MCV, fL	90.0 (86.8, 93.0)	90.5 (87.8, 93.5)	88.1 (85.1, 92.1)	89.8 (86.8, 93.0)	0.027
MCH, pg	30.90 (29.88, 31.80)	31.00 (30.10, 32.10)	30.60 (29.40, 31.30)	30.90 (29.90, 31.80)	0.024
MCHC, g/L	342 (335, 350)	341 (335, 349)	340 (334, 350)	342 (336, 350)	0.729
Cardiac biomarkers, median (IQR)					
BNP, pg/mL	196 (49, 558)	193 (59, 471)	216 (65, 471)	147 (44, 817)	0.680
Troponin, ng/mL	0.01 (0.00, 0.02)	0.01 (0.00, 0.03)	0.01 (0.00, 0.02)	0.00 (0.00, 0.01)	<0.001

[1]n (%)

© 2025. Cao R et al. Published online at http://www.aginganddisease.org/EN/10.14336/AD.2025.0540

SUPPLEMENTARY DATA

Characteristic	Cohort				P-value[2]
	Overall, N = 392[1]	Training Cohort, N = 160[1]	Internal Test Cohort, N = 69[1]	External Test Cohort, N = 163[1]	

[2]Kruskal-Wallis rank sum test; Pearson's Chi-squared test; Fisher's exact test
IQR = interquartile range; IA-tPA = intra-arterial tissue plasminogen activator; PTAS = percutaneous transluminal angioplasty and stenting; SWIM = simple wire manipulation; ALT = alanine aminotransferase; AST = aspartate aminotransferase; PT = prothrombin time; APTT = activated partial thromboplastin time; INR = international normalized ratio; RBC = red blood cell; WBC = white blood cell; MCV = mean corpuscular volume; MCH = mean corpuscular hemoglobin; MCHC = mean corpuscular hemoglobin concentration; BNP = brain natriuretic peptide

Supplementary Table 3. Relationship between baseline characteristics and outcomes.

Characteristics	Training Cohort			Internal Test Cohort			External Test Cohort		
	Effective, N = 97[1]	Futile, N = 63[1]	P-value[2]	Effective, N = 38[1]	Futile, N = 31[1]	P-value[2]	Effective, N = 88[1]	Futile, N = 75[1]	P-value[2]
**Age, years**			<0.001			0.301			0.498
Mean ± SD	68 ± 13	78 ± 11		70 ± 14	73 ± 12		61 ± 13	63 ± 14	
**Sex, n (%)**			0.110			0.989			0.191
Female	31 (32%)	28 (44%)		16 (42%)	13 (42%)		17 (19%)	21 (28%)	
Male	66 (68%)	35 (56%)		22 (58%)	18 (58%)		71 (81%)	54 (72%)	
**Intravenous thrombolysis, n (%)**			0.106			0.290			0.876
No	71 (73%)	53 (84%)		25 (66%)	24 (77%)		62 (70%)	52 (69%)	
Yes	26 (27%)	10 (16%)		13 (34%)	7 (23%)		26 (30%)	23 (31%)	
**Baseline NIHSS,**			<0.001			0.049			<0.001
Mean ± SD	10.7 ± 5.2	14.5 ± 6.5		11 ± 7	14 ± 6		13.5 ± 4.8	17.3 ± 5.2	
**Atrial fibrillation**			<0.001			0.086			0.614
No	70 (72%)	27 (43%)		25 (66%)	14 (45%)		72 (82%)	59 (79%)	
Yes	27 (28%)	36 (57%)		13 (34%)	17 (55%)		16 (18%)	16 (21%)	
**Hypertension**			0.095			0.615			0.821
No	30 (31%)	12 (19%)		9 (24%)	9 (29%)		36 (41%)	32 (43%)	
Yes	67 (69%)	51 (81%)		29 (76%)	22 (71%)		52 (59%)	43 (57%)	
**Diabetes mellitus**			0.353			0.016			0.665
No	64 (66%)	37 (59%)		28 (74%)	14 (45%)		66 (75%)	54 (72%)	
Yes	33 (34%)	26 (41%)		10 (26%)	17 (55%)		22 (25%)	21 (28%)	
**Dyslipidemia**			0.839			0.614			0.869
No	57 (59%)	36 (57%)		17 (45%)	12 (39%)		69 (78%)	58 (77%)	
Yes	40 (41%)	27 (43%)		21 (55%)	19 (61%)		19 (22%)	17 (23%)	
**Current smoking**			0.002			0.177			0.956
No	58 (60%)	52 (83%)		29 (76%)	19 (61%)		52 (59%)	44 (59%)	
Yes	39 (40%)	11 (17%)		9 (24%)	12 (39%)		36 (41%)	31 (41%)	
**Prior stroke/TIA**			0.643			0.510			>0.999
No	59 (61%)	36 (57%)		25 (66%)	18 (58%)		88 (100%)	75 (100%)	
Yes	38 (39%)	27 (43%)		13 (34%)	13 (42%)		0 (0%)	0 (0%)	
**Coronary artery disease**			0.085			0.001			0.089
No	64 (66%)	33 (52%)		28 (74%)	11 (35%)		76 (86%)	57 (76%)	
Yes	33 (34%)	30 (48%)		10 (26%)	20 (65%)		12 (14%)	18 (24%)	
**Prior anticoagulation therapy**			0.738			0.202			>0.999
No	82 (85%)	52 (83%)		34 (89%)	24 (77%)		88 (100%)	75 (100%)	
Yes	15 (15%)	11 (17%)		4 (11%)	7 (23%)		0 (0%)	0 (0%)	
**Prior antiplatelet therapy**			0.125			0.588			>0.999
No	78 (80%)	44 (70%)		28 (74%)	21 (68%)		88 (100%)	75 (100%)	
Yes	19 (20%)	19 (30%)		10 (26%)	10 (32%)		0 (0%)	0 (0%)	
**TOAST classification, n (%)**			0.173			0.124			0.576
Large-artery atherosclerosis	36 (37%)	33 (52%)		15 (39%)	14 (45%)		49 (56%)	48 (64%)	
Cardioembolism	55 (57%)	29 (46%)		22 (58%)	12 (39%)		31 (35%)	24 (32%)	
Other determined etiology	1 (1%)	0 (0%)		1 (3%)	2 (6%)		3 (3%)	1 (1%)	
Undetermined etiology	5 (5%)	1 (2%)		0 (0%)	3 (10%)		5 (6%)	2 (3%)	
**Occlusion site, n (%)**			0.133			0.023			0.210
ICA	27 (28%)	26 (41%)		11 (29%)	7 (23%)		29 (33%)	34 (45%)	
M1	41 (42%)	26 (41%)		16 (42%)	20 (65%)		47 (53%)	28 (37%)	
M2	24 (25%)	8 (13%)		10 (26%)	1 (3%)		10 (11%)	10 (13%)	
Tandem	5 (5%)	3 (3%)		1 (3%)	3 (6%)		2 (0%)	3 (0%)	
**Clot burden score**			0.185			<0.001			0.205
Mean ± SD	5.92 ± 2.83	5.22 ± 3.45		6.87 ± 2.78	4.45 ± 2.59		5.64 ± 1.78	5.27 ± 1.91	
**Whole-brain arterial collateral score**			<0.001			<0.001			<0.001

© 2025. Cao R et al. Published online at http://www.aginganddisease.org/EN/10.14336/AD.2025.0540

SUPPLEMENTARY DATA

Characteristics	Training Cohort			Internal Test Cohort			External Test Cohort		
	Effective, N = 97[1]	Futile, N = 63[1]	P-value[2]	Effective, N = 38[1]	Futile, N = 31[1]	P-value[2]	Effective, N = 88[1]	Futile, N = 75[1]	P-value[2]
Mean ± SD	3.19 ± 0.77	1.59 ± 1.23		3.42 ± 0.72	1.48 ± 1.18		2.28 ± 1.06	1.56 ± 0.99	
**ASPECTS**			0.025			0.006			0.013
Mean ± SD	7.34 ± 2.07	6.52 ± 2.32		7.74 ± 1.50	6.26 ± 2.50		7.92 ± 2.09	7.05 ± 2.30	
**Penumbra volume**			0.048			0.063			0.003
Mean ± SD	92 ± 69	115 ± 75		76 ± 73	106 ± 61		155 ± 82	195 ± 87	
**Mismactch ratio**			0.865			0.062			0.002
Mean ± SD	5.1 ± 5.0	4.8 ± 12.6		6.6 ± 6.1	3.8 ± 6.3		23 ± 28	11 ± 19	
**Superficial middle cerebral vein**			<0.001			0.005			<0.001
Mean ± SD	2.43 ± 1.29	0.87 ± 1.26		2.50 ± 1.20	1.52 ± 1.52		2.27 ± 1.37	1.05 ± 1.31	
**Sphenoparietal sinus**			<0.001			0.006			<0.001
Mean ± SD	1.76 ± 1.58	0.51 ± 1.13		1.39 ± 1.62	0.52 ± 0.93		1.20 ± 1.46	0.29 ± 0.73	
**Vein of Labbé**			<0.001			<0.001			<0.001
Mean ± SD	2.28 ± 1.34	1.25 ± 1.32		2.97 ± 0.97	1.42 ± 1.59		2.20 ± 1.23	1.41 ± 1.48	
**Vein of Trolard**			<0.001			<0.001			0.009
Mean ± SD	3.04 ± 0.76	2.25 ± 1.18		3.26 ± 0.64	2.13 ± 1.20		2.69 ± 0.96	2.19 ± 1.39	
**Whole-brain venous collateral score**			<0.001			<0.001			<0.001
Mean ± SD	9.5 ± 3.1	4.9 ± 3.2		10.1 ± 2.6	5.6 ± 3.4		8.4 ± 3.6	4.9 ± 2.9	
**Treatment modality, n (%)**			0.539			0.003			0.139
ASP	14 (14%)	14 (22%)		10 (26%)	3 (10%)		29 (33%)	20 (27%)	
IA-Tpa	8 (8%)	2 (3%)		5 (13%)	5 (16%)		0 (0%)	0 (0%)	
PTAS	26 (27%)	14 (22%)		15 (39%)	4 (13%)		16 (18%)	13 (17%)	
Solumbra	41 (42%)	28 (44%)		6 (16%)	17 (55%)		38 (43%)	29 (39%)	
SR	8 (8%)	5 (8%)		2 (5%)	2 (6%)		3 (3%)	11 (15%)	
SWIM	0 (0%)	0 (0%)		0 (0%)	0 (0%)		2 (2%)	2 (3%)	
**Imaging to puncture**			0.149			0.857			0.412
Mean ± SD	89 ± 42	100 ± 46		92 ± 50	89 ± 70		173 ± 47	178 ± 43	
**Puncture to recanalization**			0.876			0.629			0.297
Mean ± SD	102 ± 67	104 ± 67		95 ± 55	104 ± 93		127 ± 46	134 ± 41	
**Onset to arrival**			0.754			0.006			0.833
Mean ± SD	372 ± 373	351 ± 426		387 ± 372	190 ± 175		425 ± 502	411 ± 361	
**Door to imaging**			0.731			0.209			0.318
Mean ± SD	75 ± 167	90 ± 320		193 ± 666	54 ± 40		45 ± 41	57 ± 100	
**SBP**			0.201			0.144			0.422
Mean ± SD	143 ± 19	148 ± 26		143 ± 21	151 ± 24		144 ± 24	147 ± 26	
**DBP**			0.005			0.384			0.546
Mean ± SD	78 ± 13	85 ± 16		79 ± 12	82 ± 13		81 ± 17	83 ± 17	
**Glucose, mmol/L**			0.472			0.382			0.028
Mean ± SD	7.93 ± 3.26	8.41 ± 4.64		8.7 ± 3.7	9.7 ± 5.1		7.11 ± 2.77	8.22 ± 3.52	
**Total protein,g/L**			0.592			0.731			0.305
Mean ± SD	64 ± 9	63 ± 11		66 ± 8	67 ± 10		64 ± 5	65 ± 10	
**Albumin, g/L**			0.528			0.493			0.321
Mean ± SD	39.7 ± 5.7	39.1 ± 6.1		40.4 ± 5.9	39.5 ± 5.6		38.9 ± 5.3	39.5 ± 3.4	
**ALT , U/L**			0.424			0.547			0.395
Mean ± SD	18 ± 14	23 ± 48		16 ± 8	18 ± 13		25 ± 17	23 ± 12	
**AST, U/L**			0.216			0.643			0.706
Mean ± SD	20 ± 12	27 ± 43		20 ± 14	22 ± 17		25 ± 14	24 ± 12	
**Total bilirubin, µmol/L**			0.659			0.772			0.216
Mean ± SD	15 ± 10	16 ± 9		12.5 ± 5.2	12.1 ± 5.9		16 ± 13	14 ± 8	
**Direct bilirubin, µmol/L**			0.096			0.949			0.537
Mean ± SD	5.47 ± 2.72	6.94 ± 6.58		5.06 ± 2.46	5.01 ± 2.93		6.1 ± 4.4	7.8 ± 23.5	
**Creatinine, µmol/L**			0.053			0.308			0.015
Mean ± SD	74 ± 19	103 ± 113		78 ± 23	90 ± 63		92 ± 26	82 ± 25	
**Urea, mmol/L**			0.024			0.797			0.492
Mean ± SD	5.50 ± 2.18	6.64 ± 3.53		6.05 ± 4.83	6.31 ± 3.29		326 ± 105	315 ± 93	
**Uric acid, µmol/L**			0.166			0.737			0.896
Mean ± SD	337 ± 106	365 ± 136		321 ± 133	312 ± 96		284 ± 70	283 ± 73	

© 2025. Cao R et al. Published online at http://www.aginganddisease.org/EN/10.14336/AD.2025.0540

Characteristics	Training Cohort			Internal Test Cohort			External Test Cohort		
	Effective, N = 97[1]	Futile, N = 63[1]	P-value[2]	Effective, N = 38[1]	Futile, N = 31[1]	P-value[2]	Effective, N = 88[1]	Futile, N = 75[1]	P-value[2]
**Sodium, mmol/L**			0.173			0.801			0.432
Mean ± SD	140.7 ± 2.9	139.9 ± 4.6		139.51 ± 3.53	139.31 ± 2.99		140.51 ± 2.39	140.18 ± 2.88	
**Potassium, mmol/L**			0.608			0.283			0.975
Mean ± SD	3.91 ± 0.36	3.95 ± 0.59		3.91 ± 0.48	5.33 ± 7.19		4.39 ± 4.30	4.37 ± 4.66	
**D-dimer, μg/L**			0.126			0.059			0.252
Mean ± SD	478 ± 986	1,260 ± 3,923		605 ± 1,676	1,818 ± 3,130		662 ± 1,396	1,005 ± 2,238	
**PT, s**			0.046			0.543			0.563
Mean ± SD	11.45 ± 1.93	12.07 ± 1.93		11.53 ± 1.35	11.73 ± 1.28		11.65 ± 1.49	11.52 ± 1.42	
**APTT, s**			0.111			0.519			0.384
Mean ± SD	34.9 ± 12.7	32.7 ± 4.2		32.4 ± 3.0	35.6 ± 27.4		34.7 ± 18.2	37.6 ± 24.4	
**Fibrinogen, g/L**			0.889			0.150			0.805
Mean ± SD	3.38 ± 3.08	3.43 ± 1.19		4.63 ± 6.69	3.02 ± 0.93		3.98 ± 5.45	4.20 ± 5.86	
**INR**			0.378			0.825			0.484
Mean ± SD	0.98 ± 0.16	1.00 ± 0.11		1.00 ± 0.12	1.00 ± 0.11		1.01 ± 0.13	1.00 ± 0.12	
**RBC count, $\times 10^{12}$/L**			0.221			0.719			0.436
Mean ± SD	4.38 ± 0.76	4.23 ± 0.75		4.29 ± 0.79	4.36 ± 0.87		4.37 ± 0.98	4.48 ± 0.83	
**WBC count, $\times 10^{9}$/L**			0.290			0.013			0.218
Mean ± SD	8.11 ± 2.42	8.62 ± 3.27		7.96 ± 2.75	9.94 ± 3.48		10.69 ± 13.17	8.91 ± 2.86	
**Platelet count, $\times 10^{9}$/L**			0.003			0.352			0.863
Mean ± SD	215 ± 60	187 ± 53		202 ± 64	186 ± 74		211 ± 58	213 ± 61	
**Hemoglobin, g/L**			0.209			0.734			0.387
Mean ± SD	134 ± 21	130 ± 21		133 ± 21	131 ± 27		133 ± 24	130 ± 21	
**Hematocrit, %**			0.352			0.835			0.172
Mean ± SD	39.2 ± 5.7	38.3 ± 5.8		39 ± 5	38 ± 7		43.8 ± 10.7	41.8 ± 8.2	
**MCV, fL**			0.659			0.743			0.923
Mean ± SD	90 ± 6	90 ± 7		88.9 ± 5.1	88.5 ± 4.9		89.7 ± 5.0	89.6 ± 5.8	
**MCH, pg**			0.822			0.461			0.820
Mean ± SD	30.86 ± 2.38	30.77 ± 2.49		30.49 ± 1.97	30.15 ± 1.74		30.70 ± 1.92	30.78 ± 2.24	
**MCHC, g/L**			0.093			0.431			0.750
Mean ± SD	343 ± 12	340 ± 11		343 ± 13	341 ± 11		342 ± 10	343 ± 14	
**BNP, pg/mL**			0.005			0.033			0.551
Mean ± SD	239 ± 306	536 ± 773		228 ± 234	494 ± 636		588 ± 920	682 ± 1,057	
**Troponin, ng/mL**			0.622			0.259			0.277
Mean ± SD	0.267 ± 1.743	0.166 ± 0.817		0.014 ± 0.014	52.388 ± 253.504		0.18 ± 1.15	20.71 ± 162.47	

[1]n (%)
[2]Kruskal-Wallis rank sum test; Pearson's Chi-squared test; Fisher's exact test
IQR = interquartile range; IA-tPA = intra-arterial tissue plasminogen activator; PTAS = percutaneous transluminal angioplasty and stenting; SWIM = simple wire manipulation; ALT = alanine aminotransferase; AST = aspartate aminotransferase; PT = prothrombin time; APTT = activated partial thromboplastin time; INR = international normalized ratio; RBC = red blood cell; WBC = white blood cell; MCV = mean corpuscular volume; MCH = mean corpuscular hemoglobin; MCHC = mean corpuscular hemoglobin concentration; BNP = brain natriuretic peptide

Supplementary Table 4. Multiple Model Classification (Training Set)

Model	AUC (95% CI)	Cutoff (95%CI)	Accuracy (95%CI)	Sensitivity (95%CI)	Specificity (95%CI)	PPV (95%CI)	NPV (95%CI)	F1 (95%CI)	Kappa (95%CI)
XGBoost	0.988 (0.981-0.996)	0.492(0.390-0.594)	0.942(0.933-0.952)	0.932(0.905-0.959)	0.95(0.916-0.985)	0.94(0.901-0.979)	0.947(0.929-0.965)	0.935(0.926-0.944)	0.883(0.864-0.901)

© 2025. Cao R et al. Published online at http://www.aginganddisease.org/EN/10.14336/AD.2025.0540

logistic	0.862 (0.819-0.904)	0.427(0.396-0.458)	0.836(0.830-0.841)	0.821(0.797-0.844)	0.847(0.825-0.870)	0.812(0.794-0.830)	0.856(0.842-0.871)	0.816(0.810-0.821)	0.667(0.657-0.677)
LightGBM	0.967 (0.951-0.983)	0.456(0.415-0.497)	0.906(0.898-0.915)	0.894(0.869-0.920)	0.916(0.905-0.927)	0.895(0.884-0.905)	0.916(0.899-0.934)	0.894(0.883-0.906)	0.81(0.792-0.828)
Random Forest	0.850 (0.807-0.894)	0.501(0.465-0.537)	0.808(0.797-0.820)	0.717(0.643-0.790)	0.882(0.810-0.953)	0.841(0.774-0.909)	0.8(0.769-0.831)	0.767(0.752-0.783)	0.607(0.587-0.626)
SVM	0.832 (0.787-0.877)	0.387(0.352-0.422)	0.773(0.763-0.782)	0.821(0.781-0.861)	0.735(0.692-0.777)	0.713(0.686-0.740)	0.839(0.818-0.861)	0.762(0.753-0.771)	0.547(0.530-0.564)

© 2025. Cao R et al. Published online at http://www.aginganddisease.org/EN/10.14336/AD.2025.0540

SUPPLEMENTARY DATA

Supplementary Table 5. Multiple Model Classification (Test Set)

Model	AUC (95% CI)	Cutoff (95%CI)	Accuracy (95%CI)	Sensitivity (95%CI)	Specificity (95%CI)	PPV (95%CI)	NPV (95%CI)	F1 (95%CI)	Kappa (95%CI)
XGBoost	0.838 (0.745 - 0.930)	0.492(0.390-0.594)	0.793(0.754-0.832)	0.767(0.660-0.874)	0.814(0.742-0.886)	0.775(0.711-0.838)	0.822(0.753-0.890)	0.764(0.714-0.814)	0.58(0.500-0.661)
logistic	0.859 (0.773 - 0.945)	0.427(0.396-0.458)	0.828(0.806-0.849)	0.811(0.761-0.861)	0.841(0.807-0.875)	0.804(0.773-0.835)	0.85(0.818-0.881)	0.806(0.779-0.833)	0.651(0.607-0.696)
LightGBM	0.845 (0.756 - 0.935)	0.456(0.415-0.497)	0.791(0.753-0.828)	0.772(0.679-0.866)	0.806(0.746-0.865)	0.764(0.712-0.816)	0.822(0.761-0.882)	0.764(0.715-0.812)	0.576(0.498-0.654)
RandomForest	0.850 (0.763 - 0.936)	0.501(0.465-0.537)	0.781(0.752-0.809)	0.694(0.600-0.789)	0.849(0.771-0.928)	0.8(0.728-0.872)	0.782(0.740-0.824)	0.735(0.693-0.776)	0.55(0.492-0.609)
SVM	0.831 (0.741 - 0.921)	0.387(0.352-0.422)	0.754(0.729-0.779)	0.8(0.773-0.827)	0.717(0.672-0.762)	0.694(0.659-0.730)	0.818(0.798-0.839)	0.743(0.721-0.765)	0.509(0.461-0.557)

© 2025. Cao R et al. Published online at http://www.aginganddisease.org/EN/10.14336/AD.2025.0540

SUPPLEMENTARY DATA

Supplementary Table 6. Subgroup analysis of machine learning model performance in the training cohort

Subgroup	Model	AUC	Accuracy	Sensitivity	Specificity	PPV	NPV	F-beta
**Age≤65**	XGBoost	0.936	0.928	0.765	0.981	0.929	0.927	0.839
	LightGBM	0.916	0.899	0.765	0.942	0.812	0.925	0.788
	Logistic Regression	0.883	0.884	0.588	0.981	0.909	0.879	0.714
	Random Forest	0.99	0.957	0.824	1	1	0.945	0.903
	SVM	0.848	0.826	0.529	0.923	0.692	0.857	0.6
**Age＞65**	XGBoost	0.934	0.856	0.83	0.884	0.88	0.835	0.854
	LightGBM	0.907	0.862	0.83	0.895	0.89	0.837	0.859
	Logistic Regression	0.883	0.833	0.761	0.907	0.893	0.788	0.822
	Random Forest	0.974	0.914	0.875	0.953	0.951	0.882	0.911
	SVM	0.851	0.753	0.716	0.791	0.778	0.731	0.746
**Afib-negative**	XGBoost	0.952	0.89	0.796	0.938	0.867	0.9	0.83
	LightGBM	0.93	0.89	0.776	0.948	0.884	0.892	0.826
	Logistic Regression	0.911	0.855	0.612	0.979	0.938	0.832	0.741
	Random Forest	0.991	0.938	0.857	0.979	0.955	0.931	0.903
	SVM	0.916	0.862	0.735	0.927	0.837	0.873	0.783
**Afib-positive**	XGBoost	0.934	0.837	0.804	0.881	0.9	0.771	0.849
	LightGBM	0.912	0.837	0.804	0.881	0.9	0.771	0.849
	Logistic Regression	0.863	0.837	0.804	0.881	0.9	0.771	0.849
	Random Forest	0.973	0.908	0.946	0.857	0.898	0.923	0.922
	SVM	0.805	0.796	0.821	0.762	0.821	0.762	0.821
**NIHSS≤15**	XGBoost	0.933	0.876	0.758	0.946	0.893	0.869	0.82
	LightGBM	0.89	0.843	0.697	0.929	0.852	0.839	0.767
	Logistic Regression	0.865	0.826	0.576	0.973	0.927	0.796	0.71
	Random Forest	0.972	0.899	0.818	0.946	0.9	0.898	0.857
	SVM	0.827	0.803	0.591	0.929	0.83	0.794	0.69
**NIHSS＞15**	XGBoost	0.992	0.938	0.974	0.885	0.927	0.958	0.95

© 2025. Cao R et al. Published online at http://www.aginganddisease.org/EN/10.14336/AD.2025.0540

	LightGBM	0.973	0.969	0.974	0.962	0.974	0.962	0.974
	Logistic Regression	0.951	0.923	0.923	0.923	0.947	0.889	0.935
	Random Forest	0.997	0.969	0.974	0.962	0.974	0.962	0.974
	SVM	0.955	0.923	0.923	0.923	0.947	0.889	0.935
**TIA-negative**	XGBoost	0.952	0.896	0.845	0.93	0.891	0.899	0.867
	LightGBM	0.935	0.889	0.862	0.907	0.862	0.907	0.862
	Logistic Regression	0.904	0.861	0.707	0.965	0.932	0.83	0.804
	Random Forest	0.992	0.944	0.897	0.977	0.963	0.933	0.929
	SVM	0.896	0.868	0.759	0.942	0.898	0.853	0.822

Supplementary Table 7. Subgroup analysis of machine learning model performance in the external validation cohort

Subgroup	Model	AUC	Accuracy	Sensitivity	Specificity	PPV	NPV	F-beta
**Age≤65**	XGBoost	0.724	0.688	0.659	0.714	0.674	0.7	0.667
	LightGBM	0.733	0.677	0.75	0.612	0.635	0.732	0.688
	Logistic Regression	0.739	0.71	0.523	0.878	0.793	0.672	0.63
	Random Forest	0.732	0.688	0.591	0.776	0.703	0.679	0.642
	SVM	0.721	0.656	0.545	0.755	0.667	0.649	0.6
**Age＞65**	XGBoost	0.795	0.7	1	0.462	0.596	1	0.747
	LightGBM	0.848	0.743	1	0.538	0.633	1	0.775
	Logistic Regression	0.812	0.7	0.935	0.513	0.604	0.909	0.734
	Random Forest	0.804	0.729	1	0.513	0.62	1	0.765
	SVM	0.801	0.7	0.839	0.59	0.619	0.821	0.712
**Afib-negative**	XGBoost	0.792	0.725	0.746	0.708	0.677	0.773	0.71
	LightGBM	0.823	0.786	0.797	0.778	0.746	0.824	0.77
	Logistic Regression	0.834	0.794	0.763	0.819	0.776	0.808	0.769
	Random Forest	0.729	0.641	0.576	0.694	0.607	0.667	0.591
	SVM	0.781	0.733	0.847	0.639	0.658	0.836	0.741
**Afib-positive**	XGBoost	0.67	0.531	0.75	0.312	0.522	0.556	0.615
	LightGBM	0.633	0.531	0.75	0.312	0.522	0.556	0.615
	Logistic Regression	0.533	0.531	0.75	0.312	0.522	0.556	0.615
	Random Forest	0.729	0.688	0.875	0.5	0.636	0.8	0.737
	SVM	0.531	0.531	0.875	0.188	0.519	0.6	0.651
**NIHSS≤15**	XGBoost	0.801	0.744	0.875	0.685	0.553	0.925	0.677
	LightGBM	0.777	0.718	0.792	0.685	0.528	0.881	0.633

© 2025. Cao R et al. Published online at http://www.aginganddisease.org/EN/10.14336/AD.2025.0540

	Logistic Regression	0.778	0.769	0.75	0.778	0.6	0.875	0.667
	Random Forest	0.774	0.744	0.792	0.722	0.559	0.886	0.655
	SVM	0.746	0.718	0.625	0.759	0.536	0.82	0.577
**NIHSS＞15**	XGBoost	0.711	0.718	0.765	0.647	0.765	0.647	0.765
	LightGBM	0.754	0.718	0.745	0.676	0.776	0.639	0.76
	Logistic Regression	0.757	0.741	0.902	0.5	0.73	0.773	0.807
	Random Forest	0.716	0.694	0.706	0.676	0.766	0.605	0.735
	SVM	0.747	0.729	0.902	0.471	0.719	0.762	0.8
**TIA-negative**	XGBoost	0.756	0.687	0.8	0.591	0.625	0.776	0.702
	LightGBM	0.748	0.693	0.827	0.58	0.626	0.797	0.713
	Logistic Regression	0.754	0.712	0.827	0.614	0.646	0.806	0.725
	Random Forest	0.748	0.675	0.747	0.614	0.622	0.74	0.679
	SVM	0.741	0.699	0.747	0.659	0.651	0.753	0.696

Supplementary Table 18: AUC Values and 95% Confidence Intervals for Variables

**Variable**	**AUC Value**	**AUC 95% Confidence Interval**
Age	0.711	(0.629-0.793)
Whole-brain arterial collateral score	0.848	(0.782-0.913)
Whole-brain venous collateral score	0.853	(0.788-0.918)

AUC calculated using the model predictions; confidence intervals are estimated using DeLong's method.

© 2025. Cao R et al. Published online at http://www.aginganddisease.org/EN/10.14336/AD.2025.0540

SUPPLEMENTARY DATA

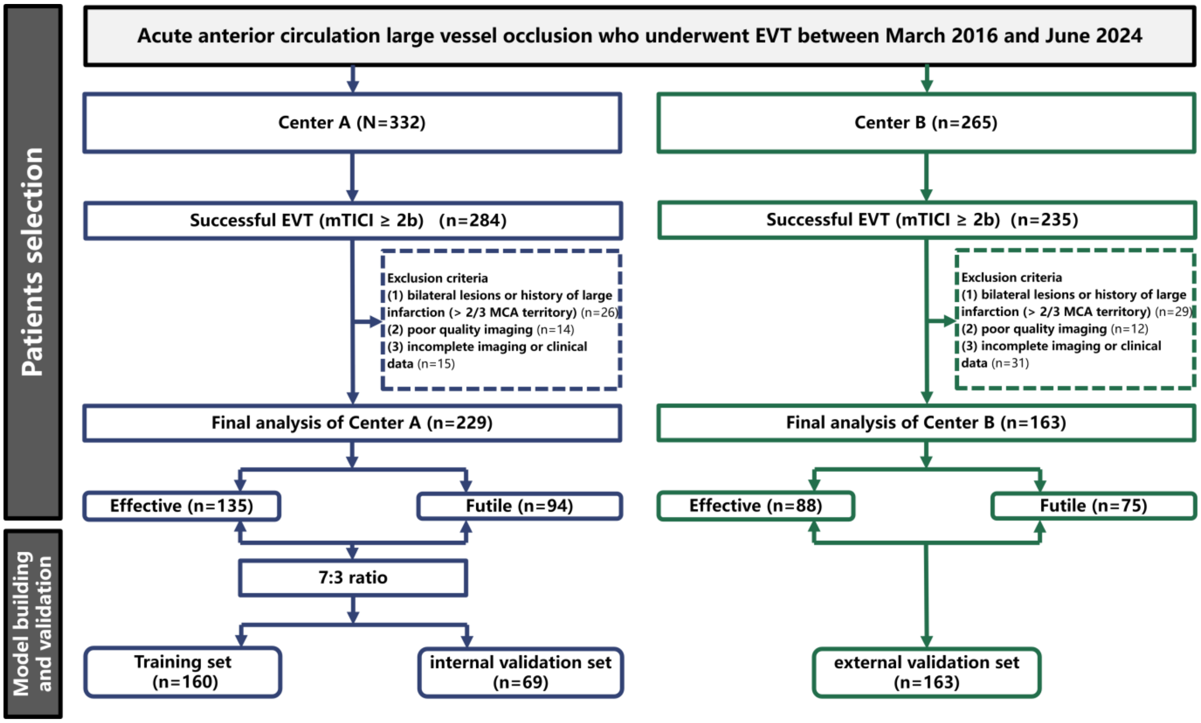

Supplementary Figure 1. Flowchart of the study population. Final cohort sizes: Training cohort n=160 (97 effective, 63 futile); Internal validation cohort n=69 (38 effective, 31 futile); External validation cohort n=163 (88 effective, 75 futile).

© 2025. Cao R et al. Published online at http://www.aginganddisease.org/EN/10.14336/AD.2025.0540

SUPPLEMENTARY DATA

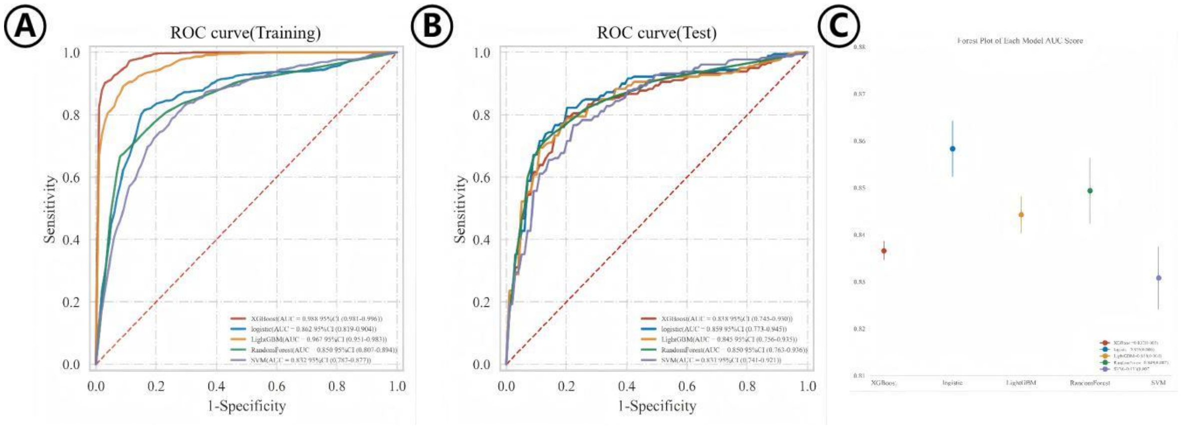

Supplementary Figure 2. The performance and comparison of five different predictive models. (A) The training set ROC curve (n=160: 97 effective, 63 futile); (B) The test set ROC curve (n=69: 38 effective, 31 futile); (C) Forest Plot of Each Model AUC Score with 95% confidence intervals calculated using DeLong's method.

© 2025. Cao R et al. Published online at http://www.aginganddisease.org/EN/10.14336/AD.2025.0540
